# A Simple Strategy for Reducing False Negatives in Calling Variants from Single-Cell Sequencing Data

**DOI:** 10.1371/journal.pone.0123789

**Published:** 2015-04-13

**Authors:** Cong Ji, Zong Miao, Xionglei He

**Affiliations:** State Key Laboratory of Biocontrol, College of Ecology and Evolution, Sun Yat-sen University, Guangzhou, 510275, China; University of North Carolina, UNITED STATES

## Abstract

Due to the growth of interest in single-cell genomics, computational methods for distinguishing true variants from artifacts are highly desirable. While special attention has been paid to false positives in variant or mutation calling from single-cell sequencing data, an equally important but often neglected issue is that of false negatives derived from allele dropout during the amplification of single cell genomes. In this paper, we propose a simple strategy to reduce the false negatives in single-cell sequencing data analysis. Simulation results show that this method is highly reliable, with an error rate of 4.94×10^-5^, which is orders of magnitude lower than the expected false negative rate (~34%) estimated from a single-cell exome dataset, though the method is limited by the low SNP density in the human genome. We applied this method to analyze the exome data of a few dozen single tumor cells generated in previous studies, and extracted cell specific mutation information for a small set of sites. Interestingly, we found that there are difficulties in using the classical clonal model of tumor cell growth to explain the mutation patterns observed in some tumor cells.

## Introduction

Multi-cellular life often starts from a single fertilized egg that develops through mitotic cell division into an organism composed of a large number of somatic cells, each of which contains an entire genome. Because DNA replication is not 100% accurate, mutations occur during every cell division, resulting in a slightly different genome for every somatic cell [1]. Similarly, cancer originates from a single somatic cell that proliferates through mitotic cell division to form a tumor composed of numerous cancer cells, each of which contains a slightly different genome [2]. It is of great interest to study such somatic mutations in single cells to understand, for instance, the effect of genetic divergence in neurons in the brain on their functional diversity or neurological disease [3], early differentiation in human embryogenesis [4], intratumoral genetic heterogeneity [5], etc. Therefore, emerging single-cell genome sequencing techniques are highly desirable research tools.

**Fig 1:**
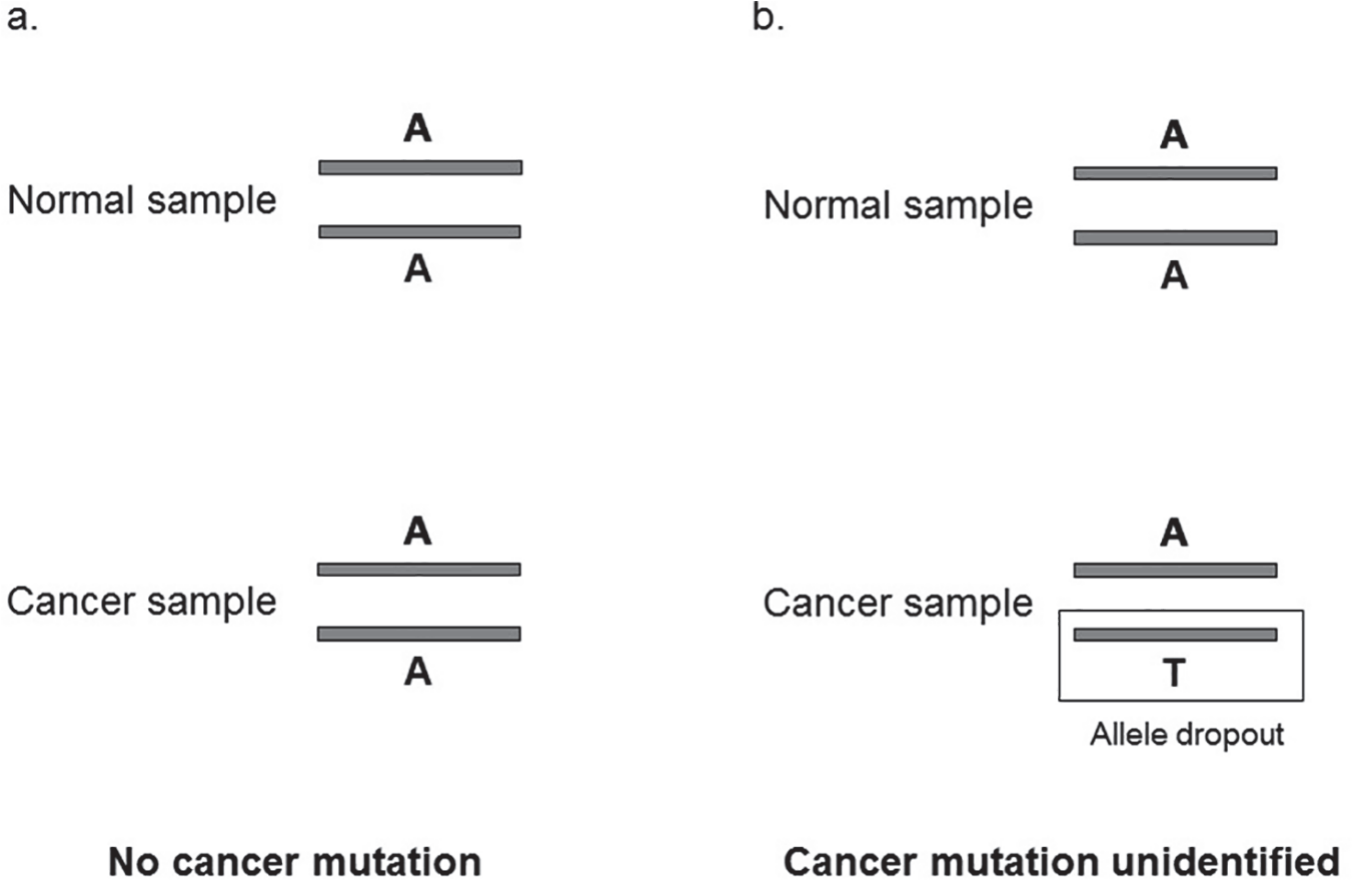
A schematic map showing how false negatives originate in cancer mutation calling. **a.** There is no cancer mutation. **b.** There is a cancer mutation that cannot be detected due to allele dropout during either single-cell genome amplification or sequencing, resulting in a false negative.

Because there are only a few copies of a gene in a cell, *in vitro* DNA amplification of the single cell’s genome is always necessary for genome sequencing. The error rate of *in vitro* DNA amplification is much higher than that of *in vivo* DNA replication, so errors that occur at early stages of amplification become a major problem in decoding a single cell’s genome [6]. Mutations are called from the sequencing reads of amplified single-cell genomes by comparing them to appropriate references, and methods for controlling false positives are often straightforward and reliable [7]. However, an intrinsic flaw of sequencing a single cell’s genome is the prevalent allele dropouts (i.e., only one of two alleles is amplified) at the early stage of genome amplification [8–12], which results in false negatives during mutation calling (Fig 1). The rate of allele dropout (ADO) used to be as high as 68% in single-cell genome amplification, and it is now reduced to 7–44% depending on the platforms used [8,11–12]. Although there were reports of significant reduction of ADO using a newly developed strategy of genome amplification [7,13], ADO remains a major confounding factor in mutation calling from single-cell genome/exome sequencing data.

In this article, we report on a method to control for false negatives due to ADO in mutation calling. Simulation results show that this method is highly reliable in reducing false negative calling errors. We applied this method to analyze the exome data of dozens of single tumor cells from previous studies [8,14], and extracted cell-specific mutational information for a small set of sites with high confidence. Interestingly, we found that there are difficulties in using the clonal growth model for tumor cells [15] to explain the mutation data in these individual cells.

## Materials and Methods

### Sequence data analysis

The raw data were downloaded from the Sequence Read Archive (SRA) websites [8,14]. It have been reported that there were three tumor cells very closing to normal cells by PCA in the original paper which may due to the pollution of extracting single cells, so we didn’t choose the data of those three cancer cells (S2 Table). The target region files of the exome captures were downloaded from the Agilent website (www.agilent.com). The reference human genome information (hg19) was downloaded from the UCSC database [16]. We aligned the pair-end reads uniquely using Bowtie2 with a 300 bp insert size [17], and found only 62 single cells from MN tumor in which 70% loci of 38M exome regions were with more than five qualified reads. But we finally chose all the 80 single cells from MN tumor to re-analyze, because it had no effect on identifying variants. Then we performed SNV identification with GATK and Picard (http://picard.sourceforge.net/). After removing PCR duplicates, we did a re-alignment around potential insertions and deletions and re-calibrated the base quality scores. We then called the SNVs by the “Unified Genotyper” mode and performed a variant quality score re-calibration. Using the standard recommended GATK filters, the SNVs located near insertions and deletions were filtered out. We only considered those loci which were covered with more than 5 qualified reads in single cells and more than 20 qualified reads in bulk tumor. This standard was still applied to compute the false negative rates. We only maintained the reads with mapping quality greater than 20, and applied a series of criteria to the target sites such as: the quality (QUAL) in the variant call format was greater than 20, the Phred scaled p value by Fisher’s exact test (FS) was less than 40 for detecting strand bias, the quality by depth (QD) was greater than 1.5 for variant confidence, and the genotype quality (GQ) was greater than 20.

### Identification of mutations

A site could be considered as a mutation only when the genotype of the target site were heterozygous in cancer samples and homozygous in normal samples. Obviously, the target sites should only be found in the exome and flanking regions within 90 bp of the mutations. Although it has been reported there were still a few data information out of exome region by the exome sequencing [18–19], here we just considered exome region. Most importantly, the target sites were excluded by the individual SNVs from normal bulk, and commonly known SNPs, including the germinal SNPs in dbsnp137 [20], the SNPs in Hapmap3.3 [21] and one thousand other genomes with minor allele frequencies greater than 0.01 [22]. The candidate mutation site was defined to be mutated in at least three tumor cells for the sufficient confidence to call a somatic mutation by a binomial distribution model considering FP as input parameter [14]. And we chose only those single cell mutations which also mutated in cancer tissues. The potentially amplified regions of the tumor genome were determined by VarScan [23], and excluded from further analyses.

### Bayesian genotype inference

We performed a Bayesian genotype inference on the SNP loci [24]. In brief, we computed the posterior probability of each genotype using the pileup qualified reads covering the locus with the following formula. First, we need to separately compute the prior probabilities *p*(*G*) of seeing these ten diploid genotypes considering GC content shown in S4 Table, which has been reported that the average GC content of the whole exome region is 41% [8]. And then we separately computed the probabilities *p*(*b*|*G*) of these ten genotypes for each base. Of note, b represented each base covering the target locus, so the probability of each base given the genotype was defined to be $p\left(b|G\right)=p\left(b|\left\{A_{1},A_{2}\right\}\right)=\frac{1}{2}p\left(b|A_{1}\right)+\frac{1}{2}p\left(b{|A}_{2}\right)$, and the genotype *G* = {*A*_1_,*A*_2_} was decomposed into two alleles. The probability of seeing a base given an allele was *p*(*b*|*A*), and the term e was the reversed Phred scaled quality score at the base. Here, it is obvious that four probabilities of four different types of bases should sum to one. It was computed by the following formula if the GC content was 50%:$p\left(b|A\right)=\{\begin{array}{cc} e/3 & \left(b\neq A\right)\\ 1-e & \left(b=A\right) \end{array}$. But if considering GC content bias with 40%, it would be computed depending on the percent of error ratio not randomly. For instance, the probability of genotype call was A if the base was C, *p*(*b*|*C*) was computed as (2/7)×*e* when GC/AT equal to 2/3. All prior probabilities when GC content was 41% [8] has been provided in S4 Table. After that, we inferred the probabilities of each ten genotypes for the specific locus by the high qualified pileup bases with the formula, where D represented our data, G represented the given genotype, *p*(*G*) was the prior probability of seeing this genotype, and *p*(*D*) was constant over all of the genotypes that could be ignored.

$$p\left(D|G\right)=\prod_{b\in pile-up}p\left(b|G\right),p\left(G|D\right)=\frac{p(G)p\left(D|G\right)}{p(D)}$$

Finally, the assigned genotype at each target locus was the genotype with the greatest posterior probability a thousand times higher than the summation of the other nine genotypes’ posterior probabilities, so if there was no such high posterior probability, we could not be sure of the genotype of the target locus.

## Results and Discussion

### A simple strategy for reducing false negatives

**Fig 2:**
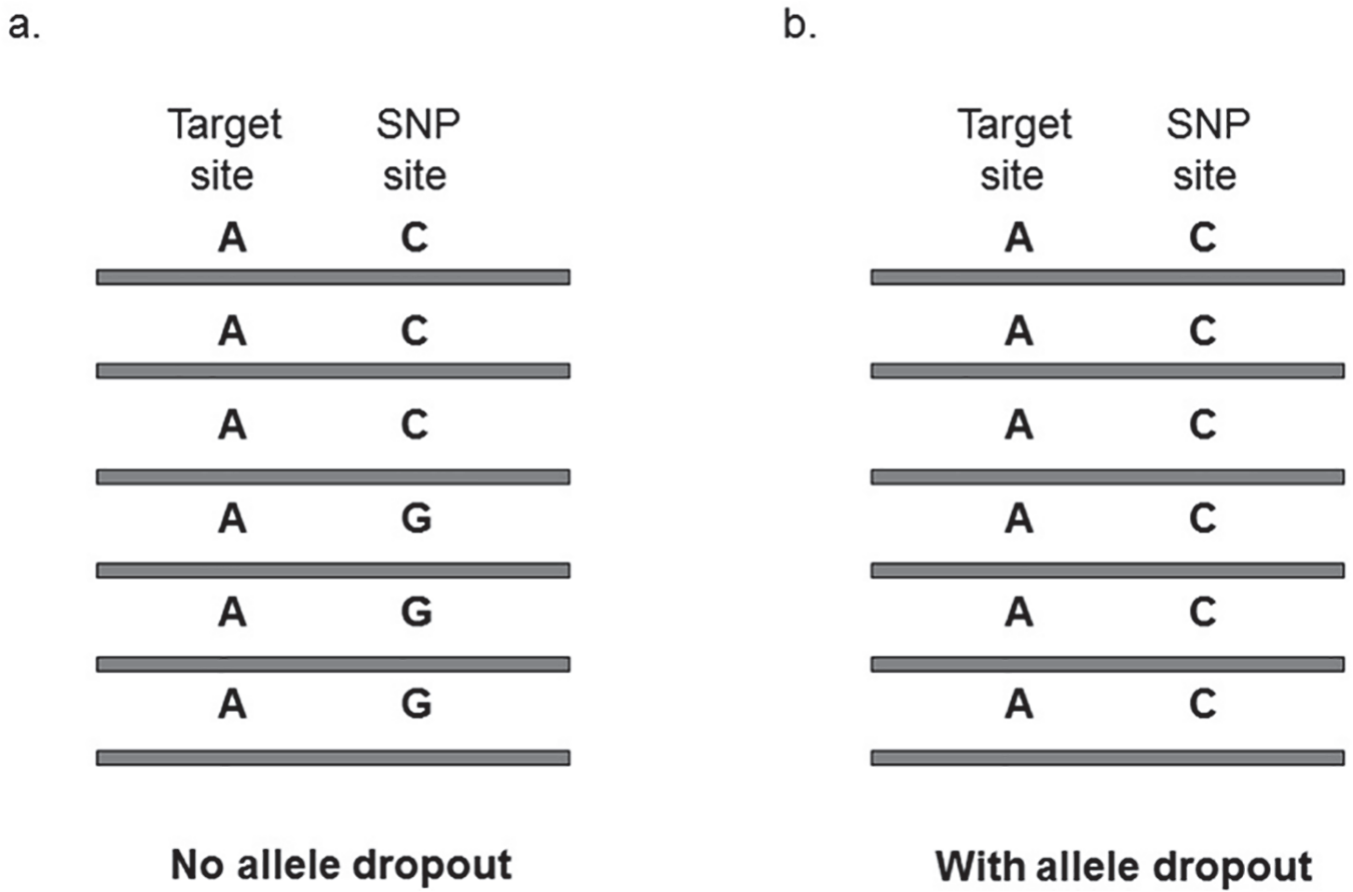
The strategy of testing whether there is allele dropout at the locus of interest. A germ-line SNP that is heterozygous in the patient and also <90 bp away from the site of interest is required. **a.** There is no allele dropout given that two alleles (such as AC, AG) are found at the SNP site. **b.** There is allele dropout given that only one allele (such as AC) is found at the SNP site.

As shown in Fig 1B, the dropout of mutant allele *T* results in only the wild-type allele *A* being available in the cancer cell; thus, this target site is mistakenly assigned to be wild type in this cancer cell, generating a false negative in mutation calling. We reasoned that neighboring polymorphisms may help to assess whether allele dropout has occurred in the region of a target site. If there is a germ-line single nucleotide polymorphic (SNP) site that is next to a target site and also heterozygous in the individual, one would expect just one allele at the SNP site when there is allele dropout, and two alleles when there is no dropout (Fig 2). Because the short reads analyzed in this study were 90 base pairs (bp) in length, we required that the SNP and target sites be separated by less than 90 bp. This way, we can obtain sequencing reads that encompass both the SNP site and the target site for our analysis. Because the two sites are tightly linked, our strategy of detecting allele dropout should be highly reliable.

### Assessment of the strategy of reducing false negatives

**Fig 3:**
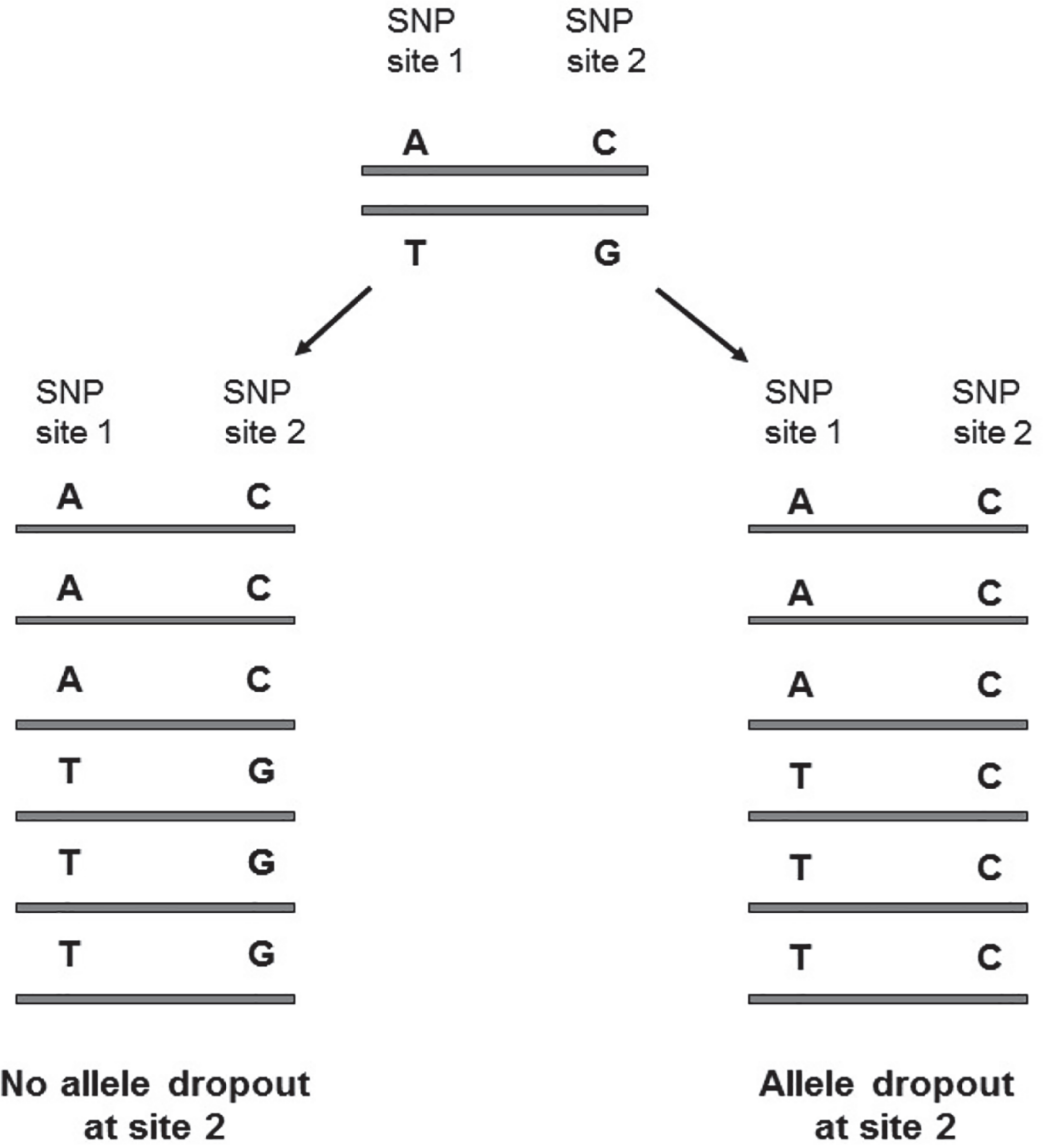
Assessing the strategy of defining allele dropout using neighboring SNPs. We considered a total of 2,320 germ-line SNP pairs in which both SNP sites were heterozygous in the patient and also <90 bp away from each other and analyzed the exome data of 80 single tumor Myeloproliferative Neoplasm cells. When there are two alleles (such as AC, TG) without allele dropout at SNP site 1, the genotype of SNP site 2 can be recovered at an error rate of 4.94×10^–5^.

To test the validity of the above strategy, the heterozygous status of a set of two SNP loci on the same reads were examined. We searched the genome for two germ-line SNP sites that are both heterozygous in the normal tissue and cancer tissue of the individual and less than 90 bp away from each other (Fig 3). The frequency of observing two alleles for one SNP site but only one allele for the other SNP site measures the error rate of our above strategy to reduce false negatives. We collected a total of 2320 such SNP pairs for our test and examined 80 single tumor cells from Myeloproliferative Neoplasm (MN) [8]. Likewise, we collected 2918 SNP pairs and examined 17 single tumor cells from kidney tumor that had available exome sequences [14]. We used a Bayesian approach to infer the genotype [24] of a site in a single tumor cell from the short sequence reads; of the 40,491 cases examined from 80 tumor cells of Myeloproliferative Neoplasm (MN) [8], we observed only two cases where one SNP site is heterozygous but the other SNP site is homozygous (or hemizygous), suggesting that the error rate of our strategy is extremely low (4.94×10^–5^). And we observed no allele dropout in the 18,587 cases examined from 17 kidney tumor cells [14]. To determine the false negative rate without using our strategy in the same data, we separately considered 36,371 SNPs in MN and 45,251 SNPs in kidney tumor that were heterozygous in both normal and cancer bulk tissues. We then computed the ratio that the heterozygous status cannot be successfully recovered from the single-cell cancer exome data, which represented the expected false negative rate. They are 34% for the MN and 27.1% for the kidney tumor (S1 Table), respectively, which are orders of magnitude higher than the rates (4.94×10^–5^, or even lower) based on our method.

### Re-analysis of the exome data in single tumor cells

**Fig 4:**
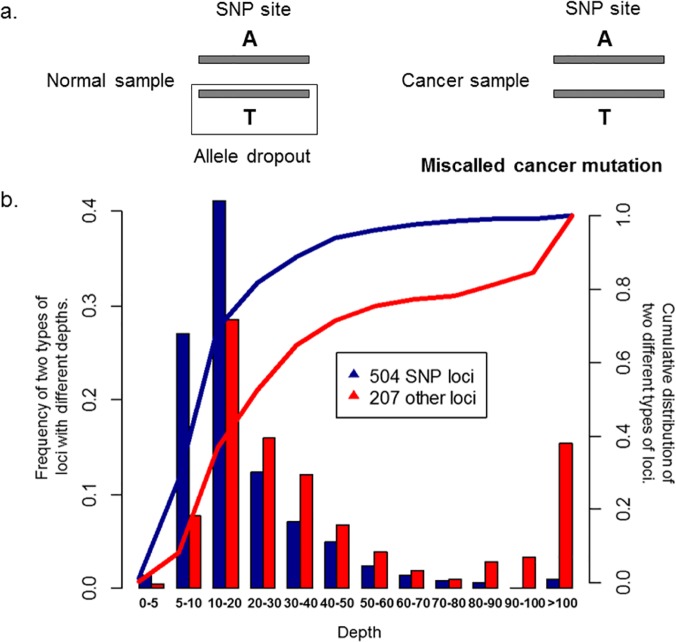
Miscalled cancer mutations in the original paper may due to allele dropout at SNP sites in the normal samples. **a.** A SNP site in a normal sample could be wrongly considered to be homozygous by allele dropout, which leaded to miscall the site in cancer samples. **b.** The frequency distribution of sequence coverage of 711 sites (504 germ-line SNP sites: blue; 207 non-SNP sites: red) in normal tissues identified in the original paper. The curves (blue for germ-line SNP sites, and red for non-SNP sites) were accumulated distribution curves.

The exome data from 17 single kidney tumor cells and 80 MN cells [8] were downloaded from two previous papers and re-analyzed (S2 Table). Using a rigorous criterion for calling mutations, we identified 343 mutations in the kidney tumor cells and 630 mutations in the MN tumor cells. About 42% (95/229) of the mutations in the kidney tumor cells detected by the previous work were recovered by our analysis, and the number is 31/35 = ~89% for the 35 mutations validated by Sanger sequencing in the previous work (S3 Table). However, only ~1% (8/711) of the mutations in the MN tumor cells detected by the previous work were also found by our method. Further examinations revealed that ~71% (504/711) of the mutations identified by the original paper [8] were corresponding to sites of common germline SNPs in human populations, with perfectly matched base identity between called mutations and annotated polymorphisms, although overall only ~0.4% (152,630/ 3.8×10^7^) of the exonic sites examined here were polymorphic (S3 Table). One explanation is that these sites, being heterozygous in the normal tissues of the patient, were mistakenly assigned to be homozygous due to low sequencing coverage in the normal tissue, and subsequent observation of heterozygosity in the tumor cells led to the erroneous calling of the mutations in the tumor cells (Fig 4A). Indeed, ~71% (360/504) of the “germline SNPs” sites were sequenced with a depth of < 20 in the normal tissue (Fig 4B and S3 Table). This result demonstrates the need of a reliable normal genotype in calling cancer mutations, as well as the importance of excluding sites corresponding to germline SNPs. In addition, there were ~11% (26/229) of the called mutations in kidney tumor cells identified by the original paper [14] corresponding to common germline SNPs.

Regardless of the apparent false positives in the two previous papers, we examined the 343 sites mutated in the kidney tumor cells and the 630 sites mutated in the MN tumor cells identified by our pipeline, to determine the exact genotypes (i.e., mutant, wild-type or uncertain) of these sites in every single tumor cell. We applied our above strategy to identify true wild-types from non-mutant genotypes, and successfully confirmed 27 true wild-types in MN cancer cells and 13 true wild-types in kidney tumor cells, respectively (Table 1 and S2 Table). Notably, the genotype of a site could be mutant in some cells, wild-type in some other cells, and uncertain in the rest tumor cells. For example, the site Chr21: 37664570 has a G->T mutation in cells LC-21, LC-16, LC-100, *et al*, and is wild-type in LC-1, LC-72 and LC-87, with uncertain genotype in other cells examined (Table 1).

**Table 1:** Details of the loci with confirmed genotype information.

PositionCell	Chr21	Chr4	Chr5	Chr6	Chr6	Chr1	Chr3	Chr6	Chr6	Chr17	Chr1	Chr19	Chr17	Chr5	Chr19	Chr16	Chr1	Chr6
	37664570	15733256	149497105	150570076	33231195	144946864	116163658	10891886	150570077	29563085	985450	3938820	2140011	149497107	35832953	66886566	157494385	90432584
	G->T	C->T	G->A	T->C	G->C	T->C	A->C	G->A	C->T	T->G	G->A	G->T	A->T	G->A	C->T	C->T	C->A	A->T
LC-1	-1	1	0	0	0	0	0	0	0	0	0	0	0	0	NA	NA	NA	NA
LC-10	0	0	0	0	1	0	0	0	0	0	0	0	0	0	NA	NA	NA	NA
LC-100	1	-1	-1	-1	-1	0	0	0	0	0	0	1	0	0	NA	NA	NA	NA
LC-11	0	0	0	0	0	0	0	0	0	0	0	0	0	0	NA	NA	NA	NA
LC-12	0	0	0	0	0	0	0	0	0	0	0	0	0	0	NA	NA	NA	NA
LC-13	0	0	0	0	0	0	0	0	0	0	0	0	0	0	NA	NA	NA	NA
LC-14	0	0	0	0	0	0	0	0	0	0	0	0	0	0	NA	NA	NA	NA
LC-15	0	0	0	0	0	0	0	0	0	0	0	0	0	0	NA	NA	NA	NA
LC-16	1	0	0	0	0	0	0	0	0	0	0	0	0	1	NA	NA	NA	NA
LC-17	0	0	0	0	0	0	0	0	0	0	0	0	0	0	NA	NA	NA	NA
LC-18	0	0	0	0	0	0	0	0	0	0	0	1	1	0	NA	NA	NA	NA
LC-19	0	0	0	0	1	-1	0	1	0	0	0	0	0	0	NA	NA	NA	NA
LC-2	0	0	0	0	0	0	0	0	0	0	0	0	0	0	NA	NA	NA	NA
LC-20	0	0	0	0	0	0	1	0	0	0	0	1	0	0	NA	NA	NA	NA
LC-21	1	0	0	0	0	0	0	0	0	0	0	0	0	0	NA	NA	NA	NA
LC-22	0	0	0	1	-1	-1	0	0	1	0	0	0	0	0	NA	NA	NA	NA
LC-23	0	0	0	0	0	0	0	0	0	0	0	0	0	0	NA	NA	NA	NA
LC-24	0	1	0	0	0	0	0	0	0	1	0	0	0	0	NA	NA	NA	NA
LC-25	0	0	0	0	0	0	0	0	0	0	0	0	0	0	NA	NA	NA	NA
LC-26	0	0	0	0	1	0	0	0	0	0	0	0	0	0	NA	NA	NA	NA
LC-27	0	0	0	0	0	1	0	0	0	0	0	0	0	0	NA	NA	NA	NA
LC-28	0	0	0	0	0	0	0	0	0	0	0	0	0	0	NA	NA	NA	NA
LC-29	1	0	0	0	0	0	0	0	0	0	0	0	0	1	NA	NA	NA	NA
LC-3	1	0	0	1	0	1	0	0	1	0	0	0	0	0	NA	NA	NA	NA
LC-30	1	1	0	0	0	0	0	0	0	0	0	0	0	0	NA	NA	NA	NA
LC-31	0	1	0	0	0	0	0	0	0	0	0	0	0	0	NA	NA	NA	NA
LC-33	0	0	0	0	0	0	0	0	0	0	0	0	0	0	NA	NA	NA	NA
LC-34	1	0	0	0	0	0	0	0	0	0	0	0	0	0	NA	NA	NA	NA
LC-35	0	1	0	-1	0	0	0	-1	-1	0	0	0	0	0	NA	NA	NA	NA
LC-36	1	0	1	0	0	1	0	0	0	0	0	0	0	1	NA	NA	NA	NA
LC-37	0	0	0	0	0	0	0	0	0	0	0	0	0	0	NA	NA	NA	NA
LC-38	0	0	0	0	0	0	0	0	0	0	0	0	0	0	NA	NA	NA	NA
LC-39	0	1	0	0	0	-1	0	0	0	0	0	0	0	0	NA	NA	NA	NA
LC-40	0	0	1	0	0	1	0	0	0	0	0	0	0	1	NA	NA	NA	NA
LC-41	0	0	1	0	0	0	0	0	0	0	0	1	0	1	NA	NA	NA	NA
LC-43	0	0	0	0	-1	0	0	0	0	0	0	0	0	1	NA	NA	NA	NA
LC-44	0	0	0	0	0	1	0	0	0	-1	0	0	0	0	NA	NA	NA	NA
LC-45	0	0	0	1	0	0	0	0	1	0	0	0	0	1	NA	NA	NA	NA
LC-47	1	0	0	1	0	0	0	0	1	0	-1	0	0	0	NA	NA	NA	NA
LC-48	0	0	0	1	1	0	0	0	1	0	0	0	0	0	NA	NA	NA	NA
LC-49	0	0	0	0	-1	-1	0	0	0	0	0	0	0	0	NA	NA	NA	NA
LC-5	1	0	0	0	0	0	0	0	0	0	0	0	0	0	NA	NA	NA	NA
LC-50	0	0	0	1	0	0	1	0	1	0	0	0	0	0	NA	NA	NA	NA
LC-52	1	0	0	1	0	-1	0	0	1	0	0	1	0	0	NA	NA	NA	NA
LC-54	1	0	0	0	1	0	1	0	0	0	0	1	1	1	NA	NA	NA	NA
LC-56	1	0	0	0	1	0	0	0	0	0	0	1	0	0	NA	NA	NA	NA
LC-6	0	0	0	0	0	0	0	0	0	0	0	0	0	0	NA	NA	NA	NA
LC-60	0	0	0	1	0	0	0	0	1	0	0	0	0	0	NA	NA	NA	NA
LC-61	0	0	0	0	1	0	0	0	0	0	0	0	0	0	NA	NA	NA	NA
LC-63	0	0	0	0	0	0	0	0	0	0	0	1	0	1	NA	NA	NA	NA
LC-66	1	0	0	1	0	0	0	0	1	0	0	1	0	0	NA	NA	NA	NA
LC-69	1	0	0	0	0	0	0	0	0	0	0	0	0	0	NA	NA	NA	NA
LC-7	0	1	0	0	-1	0	0	0	0	0	0	0	0	0	NA	NA	NA	NA
LC-70	0	0	0	0	0	1	0	0	0	0	0	0	0	0	NA	NA	NA	NA
LC-71	0	0	0	0	0	0	0	0	0	0	0	0	0	0	NA	NA	NA	NA
LC-72	-1	0	0	0	0	0	0	0	0	0	0	0	0	0	NA	NA	NA	NA
LC-73	0	0	0	0	0	0	0	0	0	0	0	0	0	0	NA	NA	NA	NA
LC-74	1	0	0	0	0	0	1	0	0	0	0	0	0	0	NA	NA	NA	NA
LC-75	1	0	0	0	0	0	0	0	0	0	0	0	0	0	NA	NA	NA	NA
LC-76	0	0	0	0	0	0	0	0	0	0	0	0	0	0	NA	NA	NA	NA
LC-77	0	0	0	0	1	0	0	0	0	0	0	1	0	0	NA	NA	NA	NA
LC-78	0	0	0	0	0	0	0	0	0	0	0	0	0	0	NA	NA	NA	NA
LC-79	0	0	0	0	-1	-1	0	0	0	0	0	0	0	0	NA	NA	NA	NA
LC-8	0	1	0	0	0	0	0	0	0	0	0	0	0	0	NA	NA	NA	NA
LC-80	1	1	0	0	1	0	0	0	0	0	0	1	-1	1	NA	NA	NA	NA
LC-81	0	0	0	0	0	0	0	0	0	0	0	1	0	0	NA	NA	NA	NA
LC-82	0	0	0	1	0	0	0	0	1	0	0	0	0	0	NA	NA	NA	NA
LC-83	0	0	0	0	0	0	0	0	0	0	0	0	0	0	NA	NA	NA	NA
LC-84	0	0	0	0	0	0	0	0	0	0	0	0	0	0	NA	NA	NA	NA
LC-85	0	0	0	0	0	0	0	0	0	0	0	0	0	0	NA	NA	NA	NA
LC-86	1	0	0	1	0	0	0	0	1	0	0	0	0	0	NA	NA	NA	NA
LC-87	-1	0	0	0	0	1	1	0	0	0	0	0	0	1	NA	NA	NA	NA
LC-88	0	0	0	0	-1	-1	0	0	0	0	0	0	0	0	NA	NA	NA	NA
LC-89	0	0	0	0	0	1	0	0	0	0	0	0	0	0	NA	NA	NA	NA
LC-9	1	1	1	0	0	0	0	0	0	0	0	0	0	1	NA	NA	NA	NA
LC-90	0	0	0	1	0	0	0	0	1	0	0	0	0	1	NA	NA	NA	NA
LC-91	0	0	0	0	0	0	0	0	0	0	0	0	0	0	NA	NA	NA	NA
LC-93	1	0	0	0	1	0	1	0	0	0	0	0	0	0	NA	NA	NA	NA
LC-94	0	0	0	0	1	0	0	0	0	0	0	0	0	0	NA	NA	NA	NA
LC-97	0	1	0	0	-1	0	0	0	0	0	0	0	0	0	NA	NA	NA	NA
RC-1	NA	NA	NA	NA	NA	NA	NA	NA	NA	NA	NA	NA	NA	NA	-1	1	0	1
RC-2	NA	NA	NA	NA	NA	NA	NA	NA	NA	NA	NA	NA	NA	NA	0	0	0	1
RC-3	NA	NA	NA	NA	NA	NA	NA	NA	NA	NA	NA	NA	NA	NA	0	0	0	0
RC-4	NA	NA	NA	NA	NA	NA	NA	NA	NA	NA	NA	NA	NA	NA	-1	-1	0	1
RC-5	NA	NA	NA	NA	NA	NA	NA	NA	NA	NA	NA	NA	NA	NA	0	1	0	0
RC-6	NA	NA	NA	NA	NA	NA	NA	NA	NA	NA	NA	NA	NA	NA	-1	1	0	1
RC-7	NA	NA	NA	NA	NA	NA	NA	NA	NA	NA	NA	NA	NA	NA	-1	1	0	1
RC-8	NA	NA	NA	NA	NA	NA	NA	NA	NA	NA	NA	NA	NA	NA	-1	1	0	0
RC-9	NA	NA	NA	NA	NA	NA	NA	NA	NA	NA	NA	NA	NA	NA	-1	1	0	0
RC-10	NA	NA	NA	NA	NA	NA	NA	NA	NA	NA	NA	NA	NA	NA	0	0	-1	0
RC-11	NA	NA	NA	NA	NA	NA	NA	NA	NA	NA	NA	NA	NA	NA	0	0	0	0
RC-12	NA	NA	NA	NA	NA	NA	NA	NA	NA	NA	NA	NA	NA	NA	-1	0	0	0
RC-13	NA	NA	NA	NA	NA	NA	NA	NA	NA	NA	NA	NA	NA	NA	-1	1	1	1
RC-14	NA	NA	NA	NA	NA	NA	NA	NA	NA	NA	NA	NA	NA	NA	-1	1	0	0
RC-16	NA	NA	NA	NA	NA	NA	NA	NA	NA	NA	NA	NA	NA	NA	-1	1	0	1
RC-18	NA	NA	NA	NA	NA	NA	NA	NA	NA	NA	NA	NA	NA	NA	0	0	0	1
RC-19	NA	NA	NA	NA	NA	NA	NA	NA	NA	NA	NA	NA	NA	NA	-1	1	1	0

**‘**LC-’ indicates MN cancer cells, and ‘RC-’ indicates kidney tumor cells. We use ‘1’ to show confirmed mutations, ‘-1’ to show confirmed wild-types, and ‘0’ to show undefined genotypes. NA means that the genotype information is not applicable.

### A mutation pattern inconsistent with the clonal expansion of tumor cells

**Fig 5:**
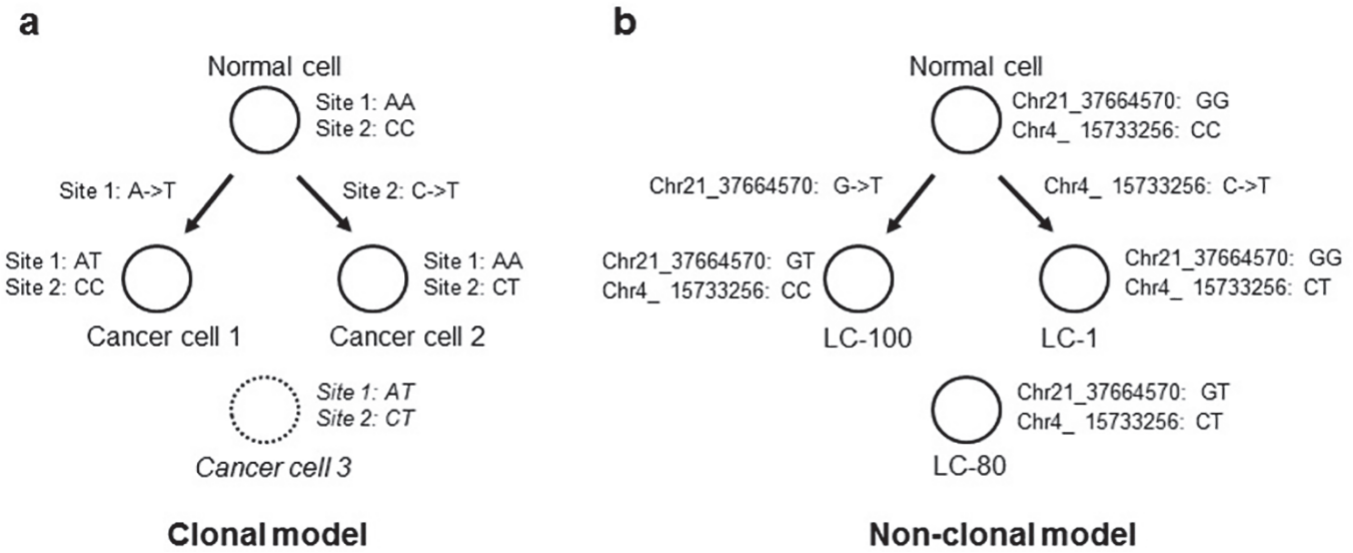
Observation of mutations that are inconsistent with the clonal model of tumor cell growth. **a.** Because recurrent mutation at the same site (Site 1 or Site 2) is unlikely, Cancer cell 3 is unforeseen according to the clonal model of tumor growth. **b.** It is difficult to explain the mutation patterns observed in the three tumor cells LC-100, LC-1, and LC-80 using the clonal model.

There is a widely held view that a tumor grows in a clonal manner through mitotic cell division [15]. According to the clonal growth model, two mutations, M1 and M2, which originate in different tumor cells will not co-exist within any cells (Fig 5A) unless there are rare recurrent mutations. An examination of the data (Table 1) revealed some interesting patterns that are summarized in Table 2. For instance, there is a G->T mutation at chr21:37664570 with a wild-type genotype at chr4:15733256 in the tumor cell LC-100, and a wild-type genotype at chr21:37664570 with a C->T mutation at chr4:15733256 in the tumor cell LC-1; unexpectedly, both mutations are found in the tumor cell LC-80 (Fig 5B). Because we define wild-type genotypes using closely linked SNPs, these types of patterns cannot be explained by a loss of heterozygosity at the regions in LC-100 or LC-1. Notably, there are quite a few such cases, suggesting that it is difficult to explain these observations based on recurrent mutations. Thus, the conventional clonal growth model seems to contradict our observations. One likely scenario is that there was a cell fusion event between the LC-100 and LC-1 lineages, resulting in the lineage of LC-80 (Fig 5B).

**Table 2:** Cases that are potentially incompatible with the clonal growth model.

**Cell ID**	**LC-1**	**LC-100**	**LC-30**
Chr21_37664570:G->T	GG	GT	GT
Chr4_15733256:C->T	CT	CC	CT
**Cell ID**	**LC-1**	**LC-100**	**LC-9**
Chr21_37664570:G->T	GG	GT	GT
Chr4_15733256:C->T	CT	CC	CT
**Cell ID**	**LC-1**	**LC-100**	**LC-80**
Chr21_37664570:G->T	GG	GT	GT
Chr4_15733256:C->T	CT	CC	CT
**Cell ID**	**LC-52**	**LC-87**	**LC-36**
Chr21_37664570:G->T	GT	GG	GT
Chr1_144946864:T->C	TT	TC	TC
**Cell ID**	**LC-52**	**LC-87**	**LC-3**
Chr21_37664570:G->T	GT	GG	GT
Chr1_144946864:T->C	TT	TC	TC

‘LC-’ indicates MN cancer cells.

## Conclusions

A major challenge in single-cell genome or exome sequencing is finding ways to call mutations accurately [6]. Previous studies have mainly been concerned with controlling false positives [7,13], so we proposed a simple method to control for false negatives by taking advantage of germ-line SNPs. While it is highly reliable, our method suffers a major limitation in that SNP density is often quite low (~0.4% of the exonic sites were polymorphic in this study), so only a small fraction of sites can be tested using this method. Nevertheless, we successfully assigned genotypes to a number of sites in single tumor cells with high confidence, and observed mutation patterns that are seemingly inconsistent with the clonal growth model of tumor cells. The amplifications may cause this observation. However, this possibility was ruled out in this study as the potentially amplified regions of the tumor genome were excluded from the analyses (see Methods). We suggest the possibility that cell fusion between different tumor cells generated the patterns. This hypothesis, if correct, has important implications for understanding tumor evolution because it suggests that mutations originating in different tumor cells can recombine with each other to select for good mutations and deplete deleterious mutations, a process similar to sexual reproduction. This being said, we are cautiously aware that the data we have are limited, and might have been biased by technical issues during the identification of the mutations in single cells. Further work is necessary to expound this hypothesis.

## Supporting Information

S1 Table The false negative rates for each single cells. To compute the false negative rates for each single cells, we collected 36371 SNPs for MN and 45251 SNPs for kidney tumor which are heterozygous in both cancer bulk and normal bulk. In order to compare with our results, we chose only loci covered with more than 5 qualified reads, and examined whether they were heterozygous in each single tumor cells or not. The false negative rates for each cells were computed by the percent of homozygous in the whole pool. We still computed the average false negative rates as follows.
(DOCX)

S2 Table The information of raw data and summary of the results. The raw data were downloaded from the Sequence Read Archive (SRA) website. It have been reported that there were three tumor cells very closing to normal cells by PCA in the original paper which may due to the pollution of extracting single cells, so we didn’t choose the data of those three cancer cells. We applied our above strategy to reduce false negatives to the 973 sites and successfully confirmed 27 wild-type genotypes for MN cancer cells and 13 wild-type genotypes for kidney tumor cells respectively.
(DOCX)

S3 Table A summary table for the comparison of variants reported by our method and previous literature. This is a summary table that displays these counts: variants discovered by our method and by the previous studies; variants only reported by our method; variants only reported by the previous studies.
(DOCX)

S4 Table The prior probabilities of ten genotypes in four specific bases if GC content is 41%. It has been reported that the average GC content of the whole exome region is 41%. And then we separately computed the probabilities *p*(*b*|*G*) of these ten genotypes for each base. The probability of seeing a base given an allele was *p*(*b*|*A*), and the term e was the reversed Phred scaled quality score at the base. Here, it is obvious that four probabilities of four different types of bases should sum to one. It was computed by the following formula if the GC content was 50%, *p*(*b*|*A*) = *e* / 3 when b is not equal to A, and *p*(*b*|*A*) = 1 – *e* when b is equal to A. But if considering GC content bias with 40%, it would be computed depending on the percent of error ratio not randomly. For instance, the probability of genotype call was A if the base was C, *p*(*b*|*C*) was computed as (2 / 7)×*e* when GC/AT equal to 2/3.
(DOCX)
