# Supplementary material for: A Simple Strategy for Reducing False Negatives in Calling Variants from Single-Cell Sequencing Data
Source: PLoS One. 2015 Apr 13;10(4):e0123789. doi: 10.1371/journal.pone.0123789 (PMC4395317; doi:10.1371/journal.pone.0123789)
Supplement: S1 Table — To compute the false negative rates for each single cells, we collected 36371 SNPs for MN and 45251 SNPs for kidney tumor which are heterozygous in both cancer bulk and normal bulk. In order to compare with our results, we chose only loci covered with more than 5 qualified reads, and examined whether they were heterozygous in each single tumor cells or not. The false negative rates for each cells were computed by the percent of homozygous in the whole pool. We still computed the average false negative rates as follows. (DOCX) [file pone.0123789.s001.docx]

**S1 Table. The false negative rates for each single cells.**

To compute the false negative rates for each single cells, we collected 36371 SNPs for MN and 45251 SNPs for kidney tumor which are heterozygous in both cancer bulk and normal bulk. In order to compare with our results, we chose only loci covered with more than 5 qualified reads, and examined whether they were heterozygous in each single tumor cells or not. The false negative rates for each cells were computed by the percent of homozygous in the whole pool. We still computed the average false negative rates as follows.

| **Cell** | **Homozygous** | **Heterozygous** | **False negative rate** |
| --- | --- | --- | --- |
| Average of MN | 34037 | 66053 | 0.340063942 |
| Average of Kidney | 9920 | 26652 | 0.271245762 |
| LC-1 | 539 | 884 | 0.378777231 |
| LC-10 | 315 | 151 | 0.675965665 |
| LC-100 | 389 | 1353 | 0.223306544 |
| LC-11 | 496 | 428 | 0.536796537 |
| LC-12 | 597 | 375 | 0.614197531 |
| LC-13 | 478 | 337 | 0.586503067 |
| LC-14 | 233 | 81 | 0.742038217 |
| LC-15 | 609 | 373 | 0.620162933 |
| LC-16 | 452 | 1159 | 0.280571074 |
| LC-17 | 314 | 102 | 0.754807692 |
| LC-18 | 502 | 756 | 0.399046105 |
| LC-19 | 587 | 681 | 0.462933754 |
| LC-2 | 460 | 401 | 0.534262485 |
| LC-20 | 610 | 666 | 0.478056426 |
| LC-21 | 546 | 478 | 0.533203125 |
| LC-22 | 446 | 1202 | 0.270631068 |
| LC-23 | 202 | 46 | 0.814516129 |
| LC-24 | 421 | 1009 | 0.294405594 |
| LC-25 | 460 | 1154 | 0.285006196 |
| LC-26 | 540 | 696 | 0.436893204 |
| LC-27 | 527 | 465 | 0.53125 |
| LC-28 | 266 | 110 | 0.707446809 |
| LC-29 | 429 | 955 | 0.309971098 |
| LC-3 | 535 | 940 | 0.362711864 |
| LC-30 | 425 | 998 | 0.298664793 |
| LC-31 | 493 | 670 | 0.423903697 |
| LC-33 | 276 | 62 | 0.816568047 |
| LC-34 | 458 | 259 | 0.638772664 |
| LC-35 | 570 | 529 | 0.518653321 |
| LC-36 | 459 | 614 | 0.4277726 |
| LC-37 | 530 | 813 | 0.394638868 |
| LC-38 | 429 | 194 | 0.688603531 |
| LC-39 | 470 | 233 | 0.6685633 |
| LC-40 | 589 | 915 | 0.39162234 |
| LC-41 | 379 | 842 | 0.31040131 |
| LC-43 | 503 | 925 | 0.352240896 |
| LC-44 | 470 | 1172 | 0.286236297 |
| LC-45 | 318 | 1400 | 0.185098952 |
| LC-47 | 318 | 1400 | 0.185098952 |
| LC-48 | 252 | 1484 | 0.14516129 |
| LC-49 | 128 | 1831 | 0.065339459 |
| LC-5 | 401 | 1193 | 0.251568381 |
| LC-50 | 411 | 1185 | 0.257518797 |
| LC-52 | 222 | 1625 | 0.120194911 |
| LC-54 | 428 | 1113 | 0.277741726 |
| LC-56 | 149 | 1729 | 0.079339723 |
| LC-6 | 394 | 1190 | 0.248737374 |
| LC-60 | 174 | 1673 | 0.094206822 |
| LC-61 | 347 | 1262 | 0.215661902 |
| LC-63 | 330 | 1229 | 0.21167415 |
| LC-66 | 232 | 1449 | 0.138013087 |
| LC-69 | 293 | 1399 | 0.173167849 |
| LC-7 | 605 | 473 | 0.56122449 |
| LC-70 | 328 | 1231 | 0.210391276 |
| LC-71 | 486 | 313 | 0.608260325 |
| LC-72 | 605 | 595 | 0.504166667 |
| LC-73 | 430 | 405 | 0.51497006 |
| LC-74 | 203 | 1577 | 0.114044944 |
| LC-75 | 423 | 276 | 0.605150215 |
| LC-76 | 548 | 512 | 0.516981132 |
| LC-77 | 540 | 553 | 0.494053065 |
| LC-78 | 565 | 706 | 0.444531865 |
| LC-79 | 177 | 1748 | 0.091948052 |
| LC-8 | 525 | 761 | 0.408242613 |
| LC-80 | 372 | 1221 | 0.233521657 |
| LC-81 | 553 | 226 | 0.709884467 |
| LC-82 | 458 | 1208 | 0.274909964 |
| LC-83 | 438 | 195 | 0.691943128 |
| LC-84 | 417 | 142 | 0.745974955 |
| LC-85 | 144 | 59 | 0.709359606 |
| LC-86 | 391 | 1339 | 0.226011561 |
| LC-87 | 465 | 999 | 0.317622951 |
| LC-88 | 587 | 624 | 0.484723369 |
| LC-89 | 548 | 815 | 0.402054292 |
| LC-9 | 587 | 715 | 0.450844854 |
| LC-90 | 558 | 813 | 0.407002188 |
| LC-91 | 462 | 794 | 0.367834395 |
| LC-93 | 410 | 1260 | 0.245508982 |
| LC-94 | 491 | 953 | 0.340027701 |
| LC-97 | 320 | 1350 | 0.191616766 |
| RC-1 | 250 | 2415 | 0.09380863 |
| RC-2 | 662 | 1515 | 0.304088195 |
| RC-3 | 685 | 1267 | 0.350922131 |
| RC-4 | 682 | 1394 | 0.328516378 |
| RC-5 | 549 | 1684 | 0.245857591 |
| RC-6 | 286 | 2405 | 0.106280193 |
| RC-7 | 360 | 2178 | 0.141843972 |
| RC-8 | 577 | 1618 | 0.262870159 |
| RC-9 | 747 | 1096 | 0.405317417 |
| RC-10 | 313 | 2099 | 0.129767828 |
| RC-11 | 778 | 967 | 0.445845272 |
| RC-12 | 693 | 1030 | 0.402205456 |
| RC-13 | 633 | 1523 | 0.293599258 |
| RC-14 | 713 | 1055 | 0.403280543 |
| RC-16 | 622 | 1764 | 0.260687343 |
| RC-18 | 573 | 1388 | 0.292197858 |
| RC-19 | 797 | 1254 | 0.388590931 |
