# Supplementary material for: A Simple Strategy for Reducing False Negatives in Calling Variants from Single-Cell Sequencing Data
Source: PLoS One. 2015 Apr 13;10(4):e0123789. doi: 10.1371/journal.pone.0123789 (PMC4395317; doi:10.1371/journal.pone.0123789)
Supplement: S2 Table — The raw data were downloaded from the Sequence Read Archive (SRA) website. It have been reported that there were three tumor cells very closing to normal cells by PCA in the original paper which may due to the pollution of extracting single cells, so we didn’t choose the data of those three cancer cells. We applied our above strategy to reduce false negatives to the 973 sites and successfully confirmed 27 wild-type genotypes for MN cancer cells and 13 wild-type genotypes for kidney tumor cells respectively. (DOCX) [file pone.0123789.s002.docx]

**S2 Table. The information of raw data and summary of the results.**

The raw data were downloaded from the Sequence Read Archive (SRA) websites ^[4] [5]^. It have been reported that there were three tumor cells very closing to normal cells by PCA in the original paper ^[5]^ which may due to the pollution of extracting single cells, so we didn’t choose the data of those three cancer cells. We applied our above strategy to reduce false negatives to the 973 sites and successfully confirmed 27 wild-type genotypes for MN cancer cells and 13 wild-type genotypes for kidney tumor cells respectively.

| **Cancer Sample** | **Myeloproliferative Neoplasm** ^[4]^ | **Kidney** ^[5]^ |
| --- | --- | --- |
| Normal Sample | Normal oral mucosal epithelium | Normal kidney |
| Number of cancer cells | 80 | 20-3= **17** |
| Number of normal cells | 7 | 5 |
| Number of single cells | 87 | 22 |
| Wide type loci | 14 | 4 |
| Wide types | **27** | **13** |
| Mutations | 114 | 20 |
| Undefined | 979 | 35 |
| Matrix | 1120 points | 68 points |
| Wide type loci × Number of cells | 14 loci ×80 cells | 4 loci ×17 cells |
