# Supplementary material for: A Simple Strategy for Reducing False Negatives in Calling Variants from Single-Cell Sequencing Data
Source: PLoS One. 2015 Apr 13;10(4):e0123789. doi: 10.1371/journal.pone.0123789 (PMC4395317; doi:10.1371/journal.pone.0123789)
Supplement: S3 Table — This is a summary table that displays these counts: variants discovered by our method and by the previous studies; variants only reported by our method; variants only reported by the previous studies. (DOCX) [file pone.0123789.s003.docx]

**S3 Table. A summary table for the comparison of variants reported by our method and by previous literature ^[4] [5]^.**

This is a summary table that displays these counts: variants discovered by our method and previous literature (separately MN ^[4]^ and kidney ^[5]^); variants only reported by our method; variants only reported by literature ^[4] [5]^.

|  | **Myeloproliferative Neoplasm** ^[4]^ | **Kidney** ^[5]^ |
| --- | --- | --- |
| **Variant Counts** |  |  |
| Discovered by our method | 630 | 343 |
| Discovered by previous literature ^[4] [5]^ | 711 | 229 |
| Shared variants | 8 | 95 |
| Only reported by our method | 622 (98.7%) | 248 (72.3%) |
| Only reported by previous literature ^[4] [5]^ | 703 (98.9%) | 134 (58.5%) |
| **Common Germline SNPs in human populations** |  |  |
| Discovered by our method | 0 | 0 |
| Discovered by previous literature ^[4] [5]^  Sequenced with a depth of < 20 in the normal tissue | 504 (70.9%)  **360 (71.4%)** | 26 (11.4%)  NA |
| **Our variants overlap with 35 PCR validated ^[5]^** | NA | 31 (88.6%) |
