# Supplementary material for: A Simple Strategy for Reducing False Negatives in Calling Variants from Single-Cell Sequencing Data
Source: PLoS One. 2015 Apr 13;10(4):e0123789. doi: 10.1371/journal.pone.0123789 (PMC4395317; doi:10.1371/journal.pone.0123789)
Supplement: S4 Table — It has been reported that the average GC content of the whole exome region is 41%. And then we separately computed the probabilities p(b|G) of these ten genotypes for each base. The probability of seeing a base given an allele was p(b|A), and the term e was the reversed Phred scaled quality score at the base. Here, it is obvious that four probabilities of four different types of bases should sum to one. It was computed by the following formula if the GC content was 50%, p(b|A) = e / 3 when b is not equal to A, and p(b|A) = 1 – e when b is equal to A. But if considering GC content bias with 40%, it would be computed depending on the percent of error ratio not randomly. For instance, the probability of genotype call was A if the base was C, p(b|C) was computed as (2 / 7)×e when GC/AT equal to 2/3. (DOCX) [file pone.0123789.s004.docx]

**S4 Table. The prior probabilities of ten genotypes in four specific bases if GC content is 41%.**

It has been reported that the average GC content of the whole exome region is 41% ^[4]^. And then we separately computed the probabilities p (b|G) of these ten genotypes for each base. The probability of seeing a base given an allele was p (b|A), and the term e was the reversed Phred scaled quality score at the base. Here, it is obvious that four probabilities of four different types of bases should sum to one. It was computed by the following formula if the GC content was 50%, p(b|A) = e/3 when b is not equal to A, and p(b|A) = 1-e when b is equal to A. But if considering GC content bias with 40%, it would be computed depending on the percent of error ratio not randomly. For instance, the probability of genotype call was A if the base was C, P(b|C) was computed as (2/7)*e when GC/AT equal to 2/3.


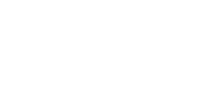


| Base  Genotype | Prior | A | T | G | C |
| --- | --- | --- | --- | --- | --- |
|  | probability |  |  |  |  |
| AA | 0.087025 | 0.9999 | 4.1844E-05 | 2.9078E-05 | 2.9078E-05 |
| TT | 0.087025 | 4.1844E-05 | 0.9999 | 2.9078E-05 | 2.9078E-05 |
| GG | 0.042025 | 3.71069E-05 | 3.71069E-05 | 0.9999 | 2.57862E-05 |
| CC | 0.042025 | 3.71069E-05 | 3.71069E-05 | 2.57862E-05 | 0.9999 |
| AT | 0.17405 | 0.499970922 | 0.499970922 | 2.9078E-05 | 2.9078E-05 |
| AG | 0.12095 | 0.499968553 | 3.94754E-05 | 0.499964539 | 2.74321E-05 |
| AC | 0.12095 | 0.499968553 | 3.94754E-05 | 2.74321E-05 | 0.499964539 |
| TG | 0.12095 | 3.94754E-05 | 0.499968553 | 0.499964539 | 2.74321E-05 |
| TC | 0.12095 | 3.94754E-05 | 0.499968553 | 2.74321E-05 | 0.499964539 |
| GC | 0.08405 | 3.71069E-05 | 3.71069E-05 | 0.499962893 | 0.499962893 |

Phred-scaled base quality = - 10 * lg(e) .

Phred-scaled base quality = ASCII of phred-scaled base quality - 33.
